# Supplementary material for: Complexome profiling on the Chlamydomonas lpa2 mutant reveals insights into PSII biogenesis and new PSII associated proteins
Source: J Exp Bot. 2021 Aug 26;73(1):245–62. doi: 10.1093/jxb/erab390 (PMC8730698; doi:10.1093/jxb/erab390)
Supplement: erab390_suppl_Supplementary_Dataset_S1 [file erab390_suppl_supplementary_dataset_s1.zip › Supplemental Dataset 1 - Excel List and all profiles/plots/_Cre06.g278222.html]

Trivial name:   
  
Euclidean distance: 20926.17  
Mean Intensity (WT): 1229.99  
Mean Intensity (Mut): 956.52  
Distance: 17.01  
  
MapMan: development.unspecified;signalling.G-proteins;signalling.receptor kinases.misc;protein.postranslational modification  
  
p value of intensity sums Welch test: 0.6958
